# Direct anterior approach versus posterolateral approach in total hip arthroplasty: a meta-analysis of results on early post-operative period

**DOI:** 10.1186/s13018-021-02218-7

**Published:** 2021-01-19

**Authors:** Xuedong Sun, Xueli Zhao, Licheng Zhou, Zheng Su

**Affiliations:** 1 Departments of Orthopaedics, Weifang People’s Hospital, no. 151 Guangwen Road, Weifang, 260041 China; 2 Department Rheumatology, Weifang People’s Hospital, no. 151 Guangwen Road, Weifang, 260041 China; 3 Department of Medical Oncology, Weifang People’s Hospital, no. 151 Guangwen Road, Weifang, 260041 China

**Keywords:** Posterolateral approach, Direct anterior approach, Total hip arthroplasty, Meta-analysis

## Abstract

**Background:**

This study was performed to compare the advantage and disadvantage of posterolateral approach (PLA) and direct anterior approach (DAA) in total hip arthroplasty (THA).

**Methods:**

Relevant trials were identified via a search of the Cochrane Central Register of Controlled Trials and PubMed from inception to 1 June 2019. A meta-analysis was performed to compare postoperative perioperative and radiographic outcomes between DAA and PLA in THA with respect to the hospital stay, blood loss, incision length, operative time, complications, and femoral and cup component position. The Harris Hip Score (HHS) was also assessed before and after 6 months postoperatively.

**Results:**

Nine eligible studies involving 22698 adult patients (DAA group, *n* = 2947; PLA group, *n* = 19751) were identified for analysis. Compared with the PLA group, the DAA group had shorter hospital stay and achieved better HHS within 6 months after operation (*P* < 0.05), but the HHS was no significant differences between the two groups over 6 months (*P* > 0.05). The DAA group had significantly longer operative time, more blood loss, and complications than the PLA group (*P* < 0.05). In addition, the femoral component positioned in neutral and cup component inclination angle was comparable between both groups (*P* > 0.05); however, cup component anteversion angle was significantly larger in the PLA group (*P* < 0.05).

**Conclusion:**

Patients in the DAA group had higher HHS within 6 months and shorter hospital stay. The DAA could offer rapid early functional recovery after THA compared with the PLA. However, the DAA group often required longer operative time and had more blood loss. Furthermore, there was a higher early complication rate. Therefore, we believe that the direct anterior approach was a more difficult technique. The surgeon should be a well-trained joint surgeon with extensive prior hip replacement experience before performing THA through a DAA, and DAA was not suitable for beginners performing THA. In addition, we did not observe the difference with regard to the femoral component position and cup component inclination angle except for the smaller cup component anteversion angle in DAA group.

## Background

Total hip arthroplasty (THA) has proven to be highly successful at alleviating pain and improving function in patients with end-stage hip arthritis. Driven by this growing demand and patients’ higher expectations, choosing the optimal surgical approach can improve the outcome of THA. The direct anterior and posterolateral approach techniques have been the subject of numerous prior investigations [1]. Proponents of the direct anterior approach (DAA) approach contend that the advantages include muscle sparing by the use of a true internervous and intermuscular plane, reduced dislocation risk, and enhanced early functional recovery [2–5]. Proponents of the posterolateral approach (PLA) pay attention to the higher rates of complications and revisions reported during the early experiences of surgeons using DAA technique [6–8]. Although some studies [9, 10] had reported similar long-term functional results between DAA and PLA in total hip arthroplasty, there is a difference in the early postoperative results. Therefore, we performed a meta-analysis of clinical studies to answer the following question: Does DAA and PLA influence the perioperative results and early functional results of a THA?

## Methods

### Search strategy

The Cochrane Central Register of Controlled Trials and PubMed databases were searched to identify relevant studies published in English from inception to 1 June 2019. The following search strategy was used to maximize search specificity and sensitivity: [THA OR THR OR (total hip)] AND [(direct anterior approach) OR DAA] AND [(posterolateral approach) OR PLA], where “THR” stands for total hip replacement.

### Selection of studies

Three independent authors (XDS, XLZ, and LCZ) initially selected studies based on their titles and abstracts. Full papers were retrieved if a decision could not be made from the titles and abstracts. Those three authors also independently assessed each full study report to see whether it met the review’s inclusion criteria. Any disagreement was discussed with the senior authors (ZS), and when consensus could not be reached, the study was excluded.

The inclusion criteria were:

- Comparison of clinical outcomes between DAA and PLA in THA
- Prospective study or retrospective study
- Cohort study, case control study, or randomized controlled trial
- Mean follow-up duration of less than 1 year
- Comparison of at least one of the following outcomes: Harris Hip Score (HHS), blood loss, hospital stay, operative time, postoperative complications, and radiographic results
- Sufficient data for extraction and pooling (i.e., reporting of the mean, standard deviation, and number of subjects for continuous outcomes and the number of subjects for dichotomous outcomes)

The exclusion criteria were:

- Revision of THA
- Review articles or case reports
- Bipolar hemiarthroplasty
- Mean follow-up duration of more than 1 year

### Data extraction

Three reviewers (XDS, XLZ, and LCZ) independently performed data extraction using standardized data extraction forms. The general characteristics of each study were extracted (i.e., Harris Hip Score (HHS), blood loss, hospital stay, operative time, postoperative complications, and radiographic results). Any disagreements were resolved by consensus or consultation with the senior authors.

### Statistical analysis

Dichotomous outcomes are expressed as the risk ratio (RR) with 95% confidence interval (CI), while continuous outcomes are expressed as the mean difference (MD) with 95% CI. Heterogeneity is expressed as *P* and *I*^2^. This value of I^2^ ranges from 0% (complete consistency) to 100% (complete inconsistency). If the *P* value of the heterogeneity test was < 0.1 or *I*^2^ > 50%, a random-effects model was used in place of the fixed modality. Publication bias was tested using funnel plots. Forest plots were used to graphically present the results of individual studies and the respective pooled estimate of effect size. All statistical analyses were performed with Review Manager (version 5.3.0 for Windows; Cochrane Collaboration, Nordic Cochrane Centre, Copenhagen, Denmark).

## Results

### Search results

**Fig. 1:**
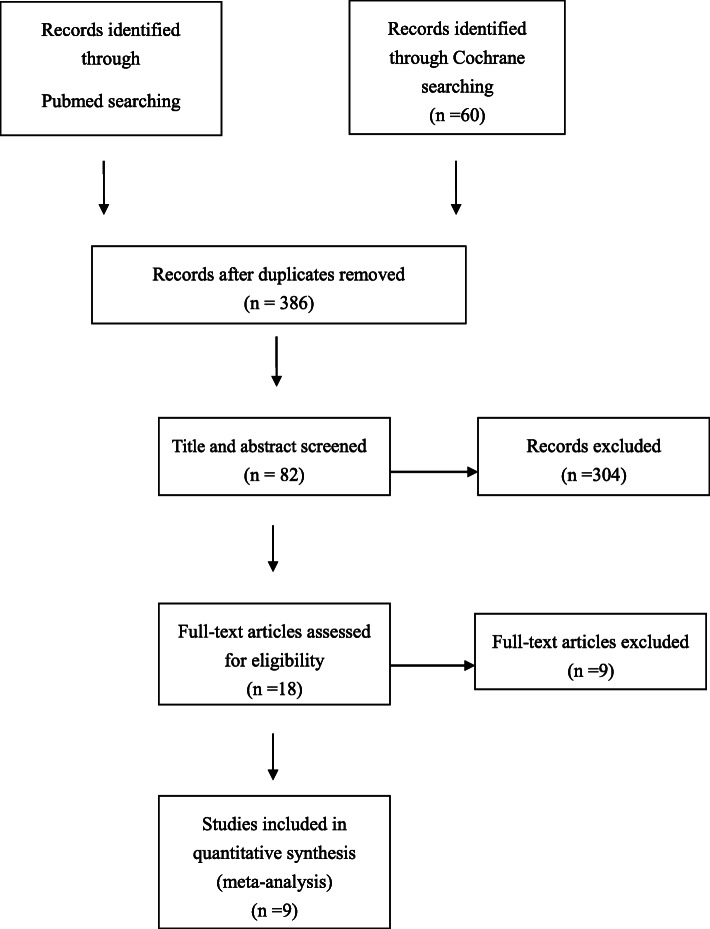
Flow of study selection

A flowchart of the studies considered for inclusion in our review is shown in Fig. 1. We identified 394 potential citations (334 from PubMed, 60 from the Cochrane Library) comparing the perioperative results and early functional results of DAA and PLA in total knee arthroplasty. After reading the articles, nine of the 394 citations were selected for the meta-analysis. The characteristics of these nine studies [11–19] are shown in Table 1.

**Table 1:** Characteristics of included studies

References	Methods	Approaches	No of patients	No of hips	Age (year)	Female/male	BMI (kg/m2)	Outcome
Barrett WP [11]	RCT	DAA	43	43	61.4	14/29	30.7	HHS, blood loss, hospital stay, operative time, complications, cup inclination angle, cup anteversion angle, femoral components position
		PLA	44	44	63.2	25/19	29.1	
Bergin PF [12]	Prospective	DAA	29	29	68.8	19/10	26.3	Hospital stay, blood loss, complications, cup inclination angle
		PLA	28	28	65.1	14/14	27.8	
Faldini C [13]	Retrospective	DAA	62	62	64	33/29	28.7	Hospital stay, operative time, complications
		PLA	65	65	65	41/24	30.1	
Fransen B [14]	Retrospective	DAA	45	45	64.2	30/15	25	Complications, cup inclination angle, femoral components position
		PLA	35	38	62.6	22/13	27.6	
Rykov [15]	RCT	DAA	23	23	62.8	15/8	29.0	HHS, hospital stay, blood loss, operative time
		PLA	23	23	60.2	12/11	29.3	
Sibia [16]	Retrospective	DAA	1457	1457	65.7	787/670	28.6	HHS
		PLA	1241	1241	65	704/537	30.4	
Spaans AJ [17]	Prospective	DAA	46	46	69	22/24	25	Hospital stay, blood loss, operative time, complications, cup inclination angle
		PLA	46	46	68	32/14	29	
Triantafyllopoulos GK [18]	Retrospective	DAA	1182	1182	62.3	626/470	NA	Hospital stay
		PLA	18213	18853	64.2	10126/8087	NA	
Zhao HY [19]	RCT	DAA	60	60	64.88	36/24	24.35	HHS, hospital stay, blood loss, operative time, complications, cup inclination angle, cup anteversion angle
		PLA	56	60	62.18	34/22	25.58	

*No* number, *DAA* direct anterior approach, *PLA* posterolateral approach, *BMI* body mass index, *HHS* Harris Hip Score, *NA* not available, *RCT* randomized controlled trial

### Meta-analysis results

**Fig. 2:**
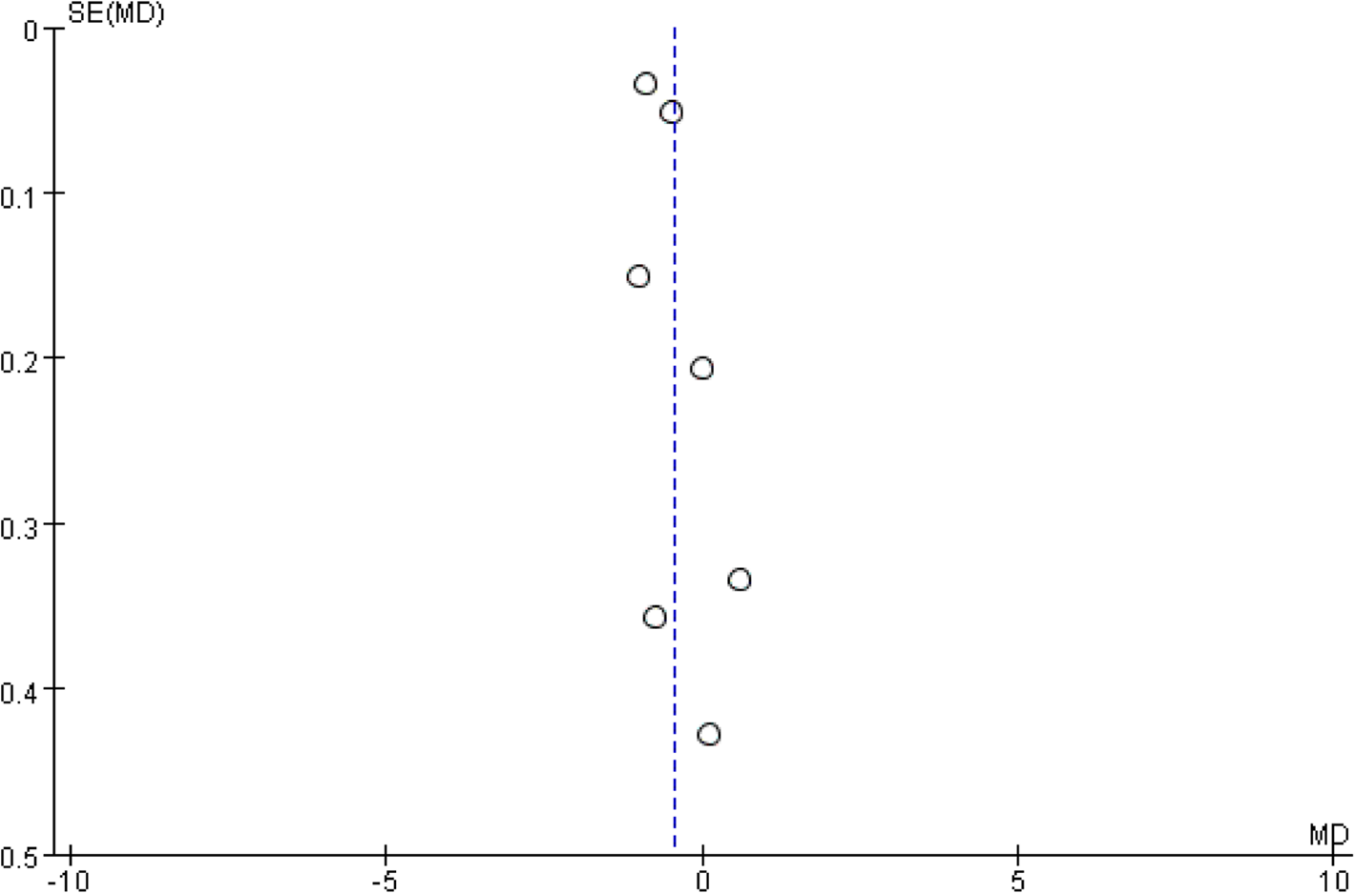
Funnel plot for hospital stay

The meta-analysis included nine studies, involving a total of 22698 patients [11–19]. The DAA group included 2947 patients, while the PLA group included 19751 patients. A funnel plot based on the most frequently cited outcome was broadly symmetrical, indicating minimal publication bias (Fig. 2).

#### HHS

**Fig. 3:**
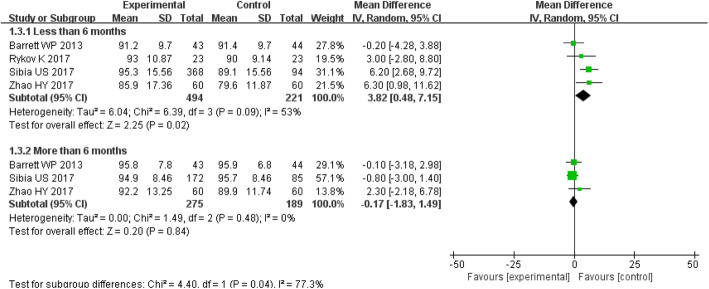
Forest plot for Harris Hip Score

The MD of the HHS within 6 months for DAA group was 3.82 (*P* = 0.02; 95% CI, 0.48–7.15), which was higher than that for PLA group. There was a significant difference between the two groups (*P* < 0.05) (Figs. 6, 7, 8). The MD of the HHS over 6 months for DAA group was − 0.17 (*P* = 0.84; 95% CI, − 1.83–1.49), No significant difference was observed between the two groups (*P* > 0.05) (Fig. 3).

#### Hospital stay

**Fig. 4:**
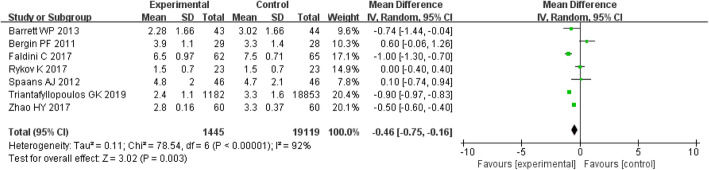
Forest plot for Hospital stay

The MD of the hospital stay for DAA group was − 0.5 (*P* = 0.003; 95% CI, − 0.6 to − 0.4), which was lower than that for PLA group. There was a significant difference between the two groups (*P* < 0.05) (Fig. 4).

#### Operative time and blood loss

**Fig. 5:**
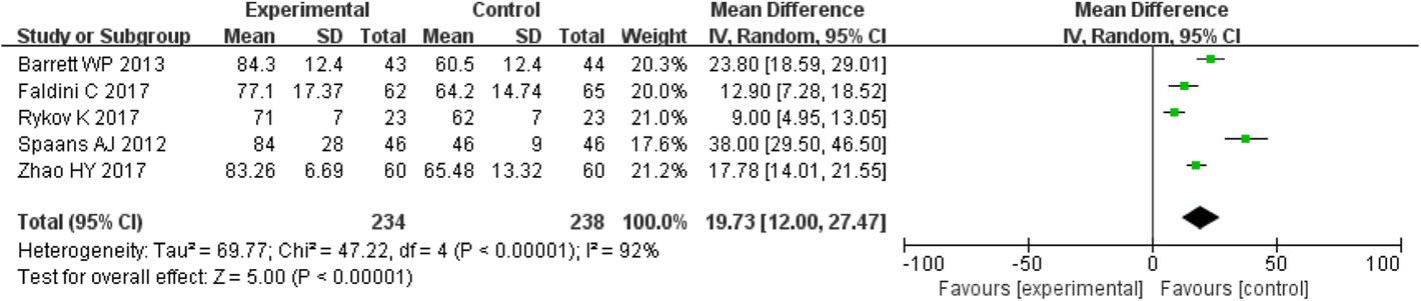
Forest plot for operative time

**Fig. 6:**
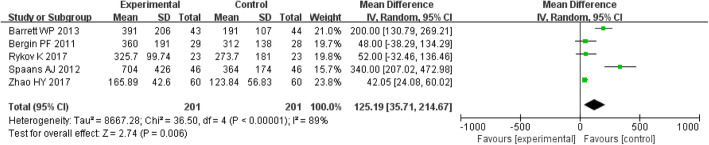
Forest plot for blood loss

The MD of the operative time and blood loss for DAA group were 19.73 (*P* < 0.00001; 95% CI, 12.00–27.47) and 125.19 (*P* = 0.006; 95% CI, 35.71–214.67), respectively, all of which were higher than those for the PLA group. The operative time and blood loss were significantly different between the two groups (*P* < 0.05) (Figs. 5 and 6).

#### Complications

**Fig. 7:**
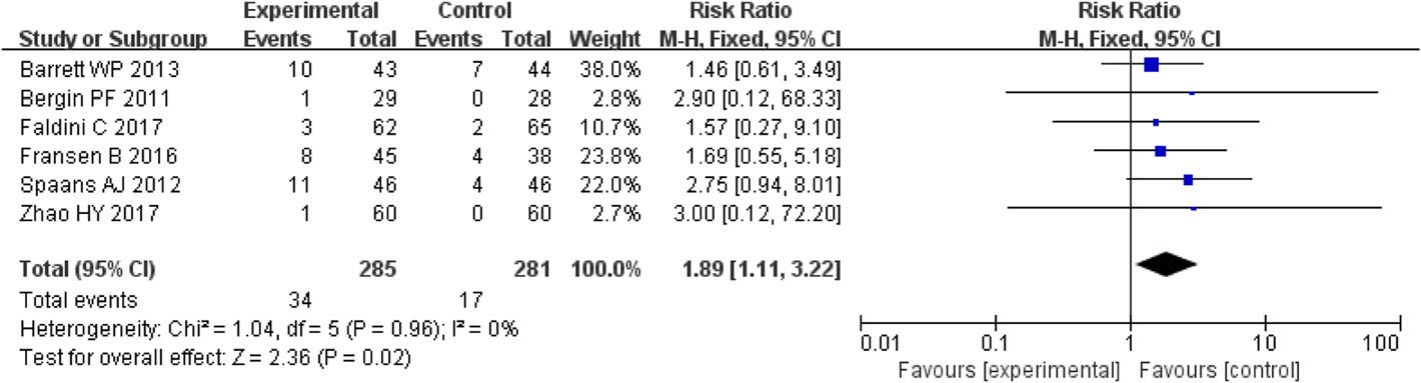
Forest plot for complications

Seven studies involving 566 patients provided data on the complications. There was a significantly greater proportion in the DAA group during the follow-up period (RR = 1.97; *P* = 0.03; 95% CI, 1.08–3.60) (Fig. 7).

#### Radiographic results

**Fig. 8:**
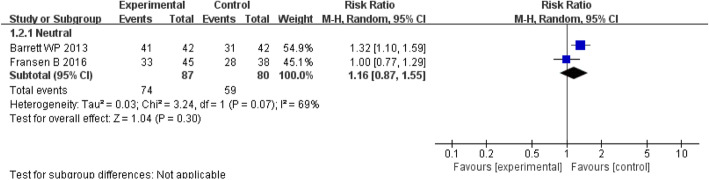
Forest plot for femoral component position

Two studies involving 133 patients provided data on femoral component position. There was a similar proportion of neutral position between the DAA group and the PLA group (RR = 1.16; *P* = 0.30; 95% CI, 0.87–1.55) (Fig. 8).

**Fig. 9:**
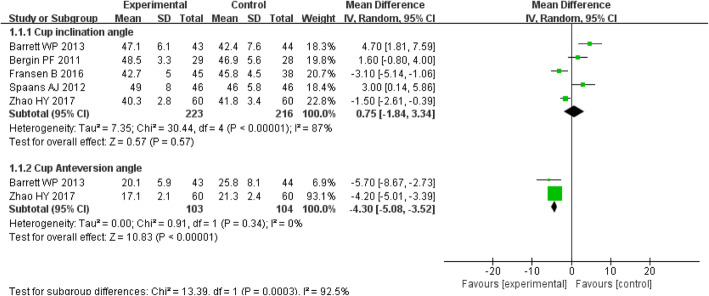
Forest plot for cup component inclination angle and anteversion angle

Five studies involving 439 patients provided data on cup component inclination angle. The MD of the inclination angle for DAA group was 0.75 (*P* = 0.57; 95% CI, − 1.84–3.34). The differences between the two groups were not statistically significant (*P* > 0.05) (Fig. 9).

Two studies involving 207 patients provided data on cup component anteversion angle. The MD of the anteversion angle for DAA group was − 4.30 (*P* < 0.00001; 95% CI, − 5.08 to − 3.52). The cup component anteversion angle was significantly smaller in the DAA group compared with the PLA group (*P* < 0.05) (Fig. 9).

## Discussion

The most important finding of the present meta-analysis was that the DAA group showed rapid early functional recovery than the PLA group. There was a greater proportion of complications in the DAA group than in the PLA group, and the DAA group often required longer operative time and had more blood loss during the operation. However, there were no significant differences between the DAA group and the PLA group in the femoral component position and cup component inclination angle except for cup component anteversion angle.

The HHS is often used to evaluate function of hip join. In our review, the DAA group yielded superior HHS within 6 months and shorter hospital stay compared with the PLA group; however, no significant difference was observed between two groups over 6 months, which is in accordance with other studies [11, 16, 19]. Some studies also reported that the DAA bring about a better stair climbing ability [20], improvement in a large number of gait parameters [21] and a more rapid recovery of hip function [22] in the short term. In addition, another advocated advantage is the significantly shorter hospital stay in the DAA group in the present study, which is in accordance with other studies. The DAA follows internervous and intermuscular planes, specifically the anatomic interval between the tensor fasciae latae and the Sartorius muscles [23, 24]. It could be considered a minimally invasive approach and theoretically result in less tissue damage than the more invasive PLA [25]. Therefore, these might indicate that the short-term benefits of the DAA continued to be reflected in superior function, but disappear after the period of 6 months.

In our study, longer operative time and more blood loss were found in patients who received THA through a DAA. The use of DAA significantly increased blood loss and operative time by a mean of 125.19 ml and 19.73 min separately. Barrett et al. [11] and Spaans et al. [17] also reported longer operative time and more blood loss in the DAA group than those in the PLA group, whereas Bergin et al. [12] and Rykov et al. [15] stated that the blood loss was no difference between the two groups; however, the operative time spent in the DAA group remained significantly extended. Early postoperative complications included trochanteric fracture, hematoma, lateral femoral cutaneous nerve damage, dislocation, persisting pain, leg length discrepancy, and deep vein thrombosis. In our study, there was a greater proportion of complications in the DAA group than in the PLA group. Spaans et al. [17] also found the similar result on complication rate. However, in some recent literature [13, 14], the complication rate in the DAA group was not significantly higher than that in the PLA group.

In the present meta-analysis, we found all the surgeons who performed THA with DAA had extensive experience. However, higher complication rate, longer operative time, and more blood loss were still found in DAA group. This indicates that the THA through a DAA was more difficult. The above pooled results were still less than satisfactory even though the surgeons involved in our study had excellent surgical technique and extensive experience. In addition, some studies [26, 27] reported that prolonged operative times were associated with an increased risk of surgical site infection. Every new operation technique is associated with effort and often with a temporary increase in adverse events, the so-called learning curve [28, 29]. Therefore, surgeon should be a well-trained joint surgeon with extensive prior hip replacement experience before performing THA through a DAA. In summary, we deemed that DAA was not suitable for beginners performing THA. This is an important conclusion we draw from this review.

The orientation of the acetabular component influences the function and durability of THA implants [30, 31]. Improper cup alignment increases risk of implant dislocation [32]. The positioning of the femoral components can influence the survival of the prosthesis [33]. In our study, the result of cup inclination angle was no significant difference between the two groups, but the result of cup anteversion angle in DAA group was significantly reduced by a mean of 4.3°. We also found the results of acetabular component position were difference among the included studies, but the mean cup inclination and anteversion angle were almost within Lewinnek’s safe zone [34] regardless of DAA group and PLA group. In addition, the position of femoral components was comparable between both groups, with similar proportion of the stems positioned in neutral. Therefore, we believe, whether it was a DAA or a PLA, the operation was satisfactory from radiographic aspect.

The limitations include the insufficient sample size and different types of prostheses used. Future studies with large sample sizes could provide enhanced analyses, and additional evaluation criteria are needed.

## Conclusion

Patients in the DAA group had higher HHS within 6 months and shorter hospital stay. The DAA could offer rapid early functional recovery after THA compared with the PLA. However, the DAA group often required longer operative time and had more blood loss. Furthermore, there was a higher early complication rate. Therefore, we believe that the direct anterior approach was a more difficult technique. The surgeon should be a well-trained joint surgeon with extensive prior hip replacement experience before performing THA through a DAA, and DAA was not suitable for beginners performing THA. In addition, we did not observe the difference with regard to the femoral component position and cup component inclination angle except for smaller cup component anteversion angle in DAA group.

## Data Availability

All data generated or analyzed during this study are included in this published article.

## Abbreviations

THA: Total hip arthroplasty
DAA: Direct anterior approach
PLA: Posterolateral approach
HHS: Harris Hip Score
RR: Risk ratio
CI: Confidence interval
MD: Mean difference
